# Breaking Free: Managing Trapped Lung in Tubercular Empyema

**DOI:** 10.7759/cureus.77971

**Published:** 2025-01-25

**Authors:** Jay Bhanushali, Babaji Ghewade, Ulhas Jadhav, Bingu Shiv Kiran Reddy, Nikhil Agarwal, Arman Sindhu

**Affiliations:** 1 Respiratory Medicine, Jawaharlal Nehru Medical College, Datta Meghe Institute of Higher Education and Research, Deemed to be University, Wardha, IND; 2 Pathology and Laboratory Medicine, Excel Diagnostics, Pune, IND

**Keywords:** decortication, empyema, pleural effusion, pleural thickening, tuberculosis

## Abstract

Tuberculosis (TB) remains a significant global health concern, particularly in high-burden countries. Pleural involvement, such as pleural thickening and trapped lung, is a common complication of tuberculous empyema, often leading to respiratory compromise and reduced quality of life. In this case report, a 58-year-old male farmer presented with progressive breathlessness and orthopnea, indicative of pleural pathology. Diagnostic thoracocentesis confirmed an exudative effusion positive for *Mycobacterium tuberculosis* complex using the cartridge-based nucleic acid amplification test (CBNAAT), which is a molecular test that can detect *M. tuberculosis* in two hours only. Prompt initiation of anti-TB therapy (anti-Koch’s treatment (AKT)) and intercostal drain insertion were performed to manage the tuberculous empyema. Imaging studies revealed pleural thickening and trapped lung, necessitating further intervention. The patient underwent lung decortication, involving extensive removal of fibrous pleural tissue, which resulted in symptomatic improvement. After the procedure, the patient successfully weaned off mechanical ventilation and achieved near-complete resolution over time. This case highlights the effective management of pleural thickening and trapped lung resulting from tuberculous empyema. The timely initiation of AKT, along with interventional procedures like lung decortication, can lead to significant improvement in symptoms and quality of life for patients with TB-related pleural complications. By addressing these complications promptly, healthcare providers can mitigate disability-adjusted life years (DALYs) associated with TB, particularly in regions with a high burden of the disease.

## Introduction

Tuberculosis (TB) remains a critical global health issue, especially in countries with high rates of infection where the disease's impact is most severe. Among the various complications of TB, pleural involvement, including conditions like tuberculous empyema (TE) and trapped lung, presents unique challenges. These complications can lead to severe respiratory problems, reduced lung function, and a noticeable decline in quality of life. TE is characterized by the buildup of pus in the pleural space. It can lead to the lung being encased in fibrous tissue, resulting in a "trapped lung" that cannot fully expand due to restrictive pleural disease [1,2].

This case report focuses on a 58-year-old farmer who experienced worsening shortness of breath and difficulty breathing while lying down, symptoms that were traced back to TE. Our approach combined prompt diagnostic measures, the initiation of anti-TB medications, and necessary surgical intervention, including lung decortication. This case underscores the critical importance of early diagnosis and timely treatment in preventing severe complications associated with TE [3,4].

By highlighting the coordinated care provided to this patient, we demonstrate how a comprehensive, multidisciplinary approach can lead to marked improvements in clinical outcomes. Effective treatment not only alleviates symptoms but also helps restore lung function and improve the patient's overall quality of life. This report serves as a stark reminder of the persistent threat of TB and underscores the need for timely and effective management, particularly in high-burden regions [5].

## Case presentation

A 58-year-old male farmer, presenting with a two-month history of progressive breathlessness and orthopnea, arrived at the emergency department. Breathlessness was insidious in onset and gradually progressive to grade 3 of the modified Medical Research Council (mMRC) scale, associated with occasional cough with expectoration. The patient did not have any known comorbidities; he had received symptomatic treatment and antibiotics from the general physician, which did not give relief, and details of the medications received were not available. Vital signs revealed a pulse rate of 117/min, respiratory rate of 24/min, blood pressure of 110/70 mmHg, and temperature of 98.7°C. Central nervous, cardiovascular, and gastrointestinal system examinations showed no abnormalities. Arterial blood gas analysis indicated a normal acid-base balance on room air.

**Figure 1:**
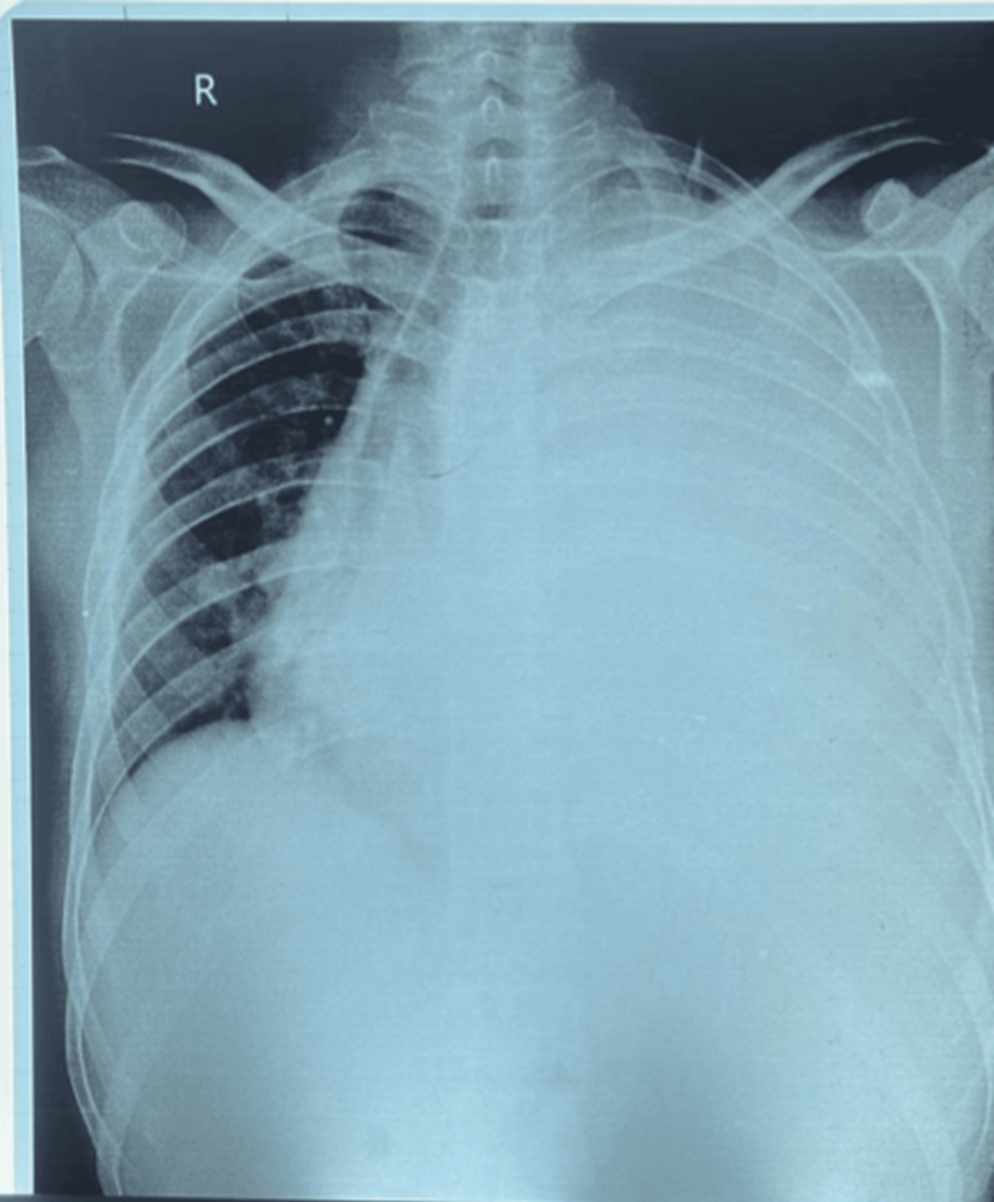
Chest X-ray PA erect showing gross left-sided pleural effusion with mediastinal shift

Respiratory system examination revealed left-sided decreased breath sounds on auscultation. A chest X-ray was done, which suggested left-sided pleural effusion with mediastinal shift (Figure 1).

**Figure 2:**
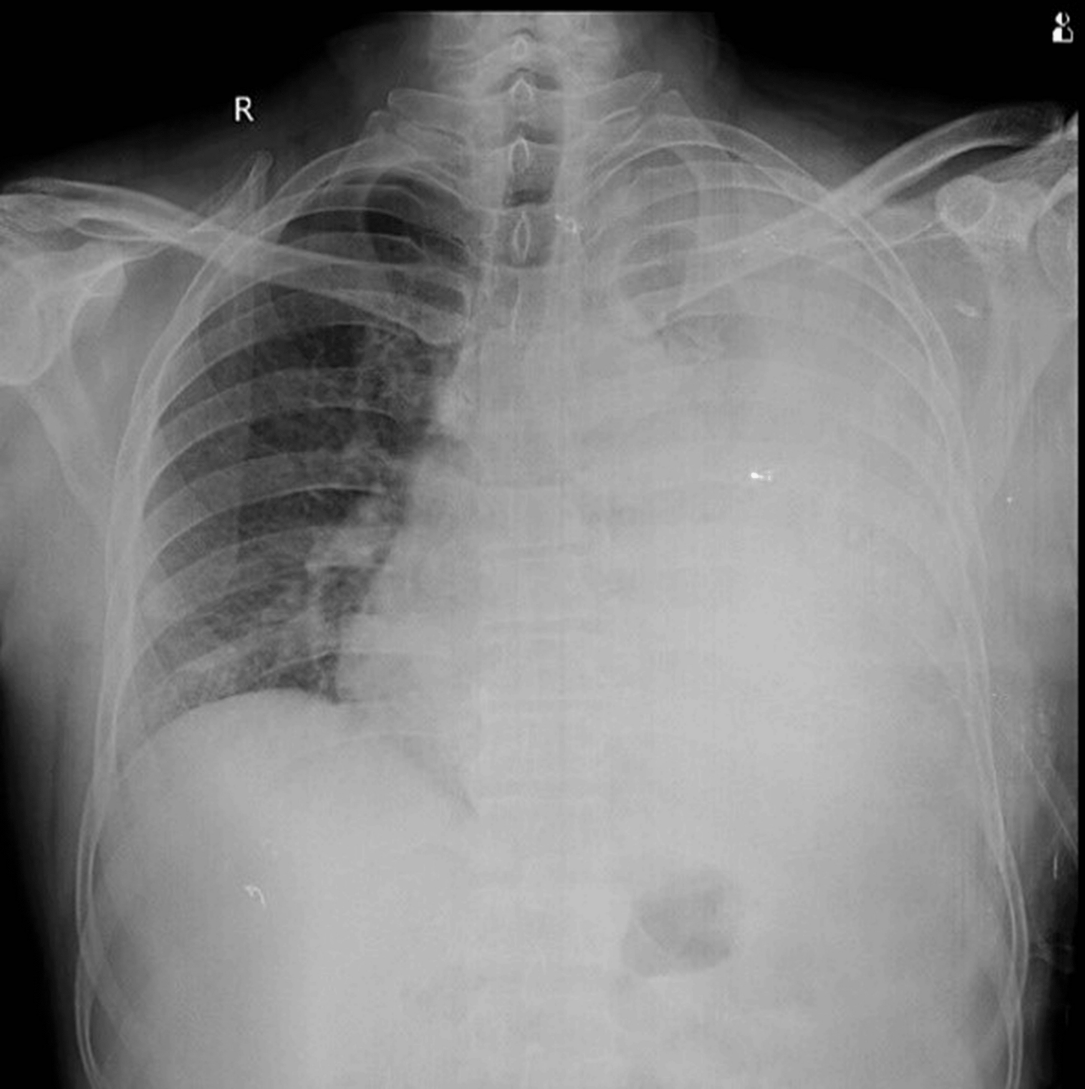
Chest X-ray post-intercostal tube insertion

Diagnostic thoracentesis was done, which revealed an exudative effusion and was positive for *Mycobacterium tuberculosis* on cartridge-based nucleic acid amplification testing (CBNAAT). The patient was started on anti-Koch’s treatment (AKT) as per the National Tuberculosis Elimination Program (NTEP) guidelines. In view of gross effusion with mediastinal shift and respiratory distress, an intercostal drain was inserted, as seen in Figure 2. 

**Figure 3:**
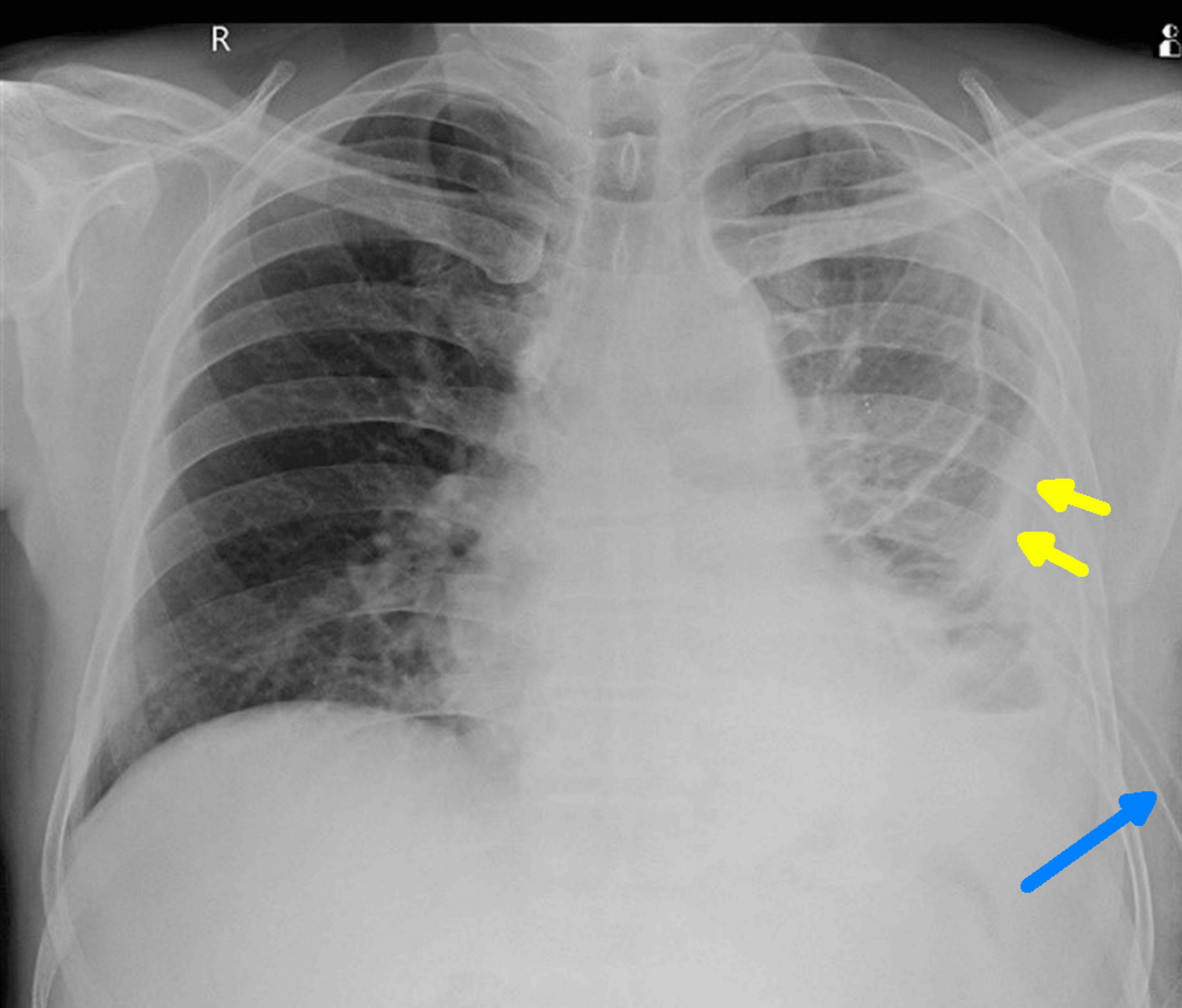
Chest X-ray post-thoracocentesis showing pleural thickening (yellow arrows) and intercostal drain in situ (blue arrow)

Around 1,200 mL of pleural fluid was drained, which was yellow-colored, had a turbid appearance, and was foul-smelling, suggestive of empyema thoracis. Post-therapeutic thoracocentesis chest X-ray was suggestive of an extensive pleural thickening and volume loss of the left hemithorax, as seen in Figure 3.

**Figure 4:**
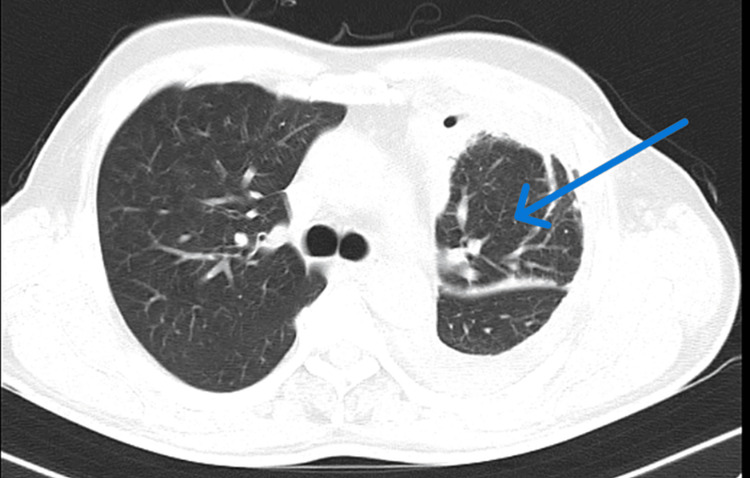
CT thorax of the chest; lung window showing trapped lung (blue arrow) CT: computed tomography

**Figure 5:**
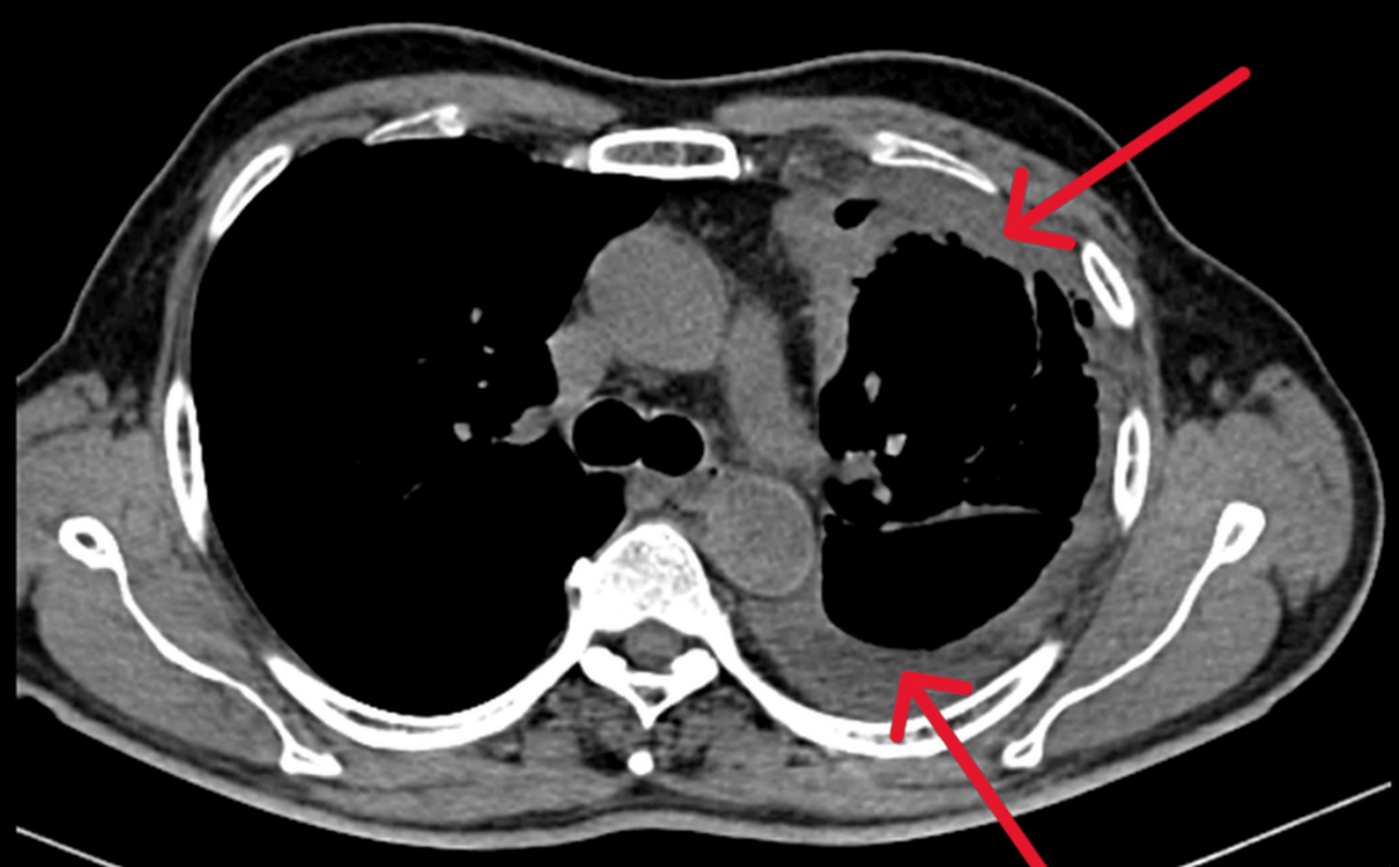
CT scan of thorax suggestive of pleural thickening (red arrows) CT: computed tomography

Subsequent computed tomography (CT) scans further supported the diagnosis of pleural thickening and trapped lung. A CT image of the chest showed pleural thickening and trapped lung as seen in Figures 4, 5.

Prompt initiation of anti-tubercular treatment was followed by lung decortication with the goal of removing fibrous pleural tissue to restore lung expansion. Informed consent for surgery was taken.

During the procedure, turbid pus was drained, and a thick layer of pleura was visualized and dissected, revealing the entrapped lung. Extensive efforts were made to excise as much fibrous pleural tissue as possible, resulting in the removal of 400 g of disease pleura.

Post-operatively, the patient received intravenous antibiotics, AKT, and other supportive medications. Within 48 hours of the operation, the patient was successfully weaned off mechanical ventilation. Antibiotic treatment included injection of cefoperazone and sulbactam 1.5 g twice daily for seven days.

**Figure 6:**
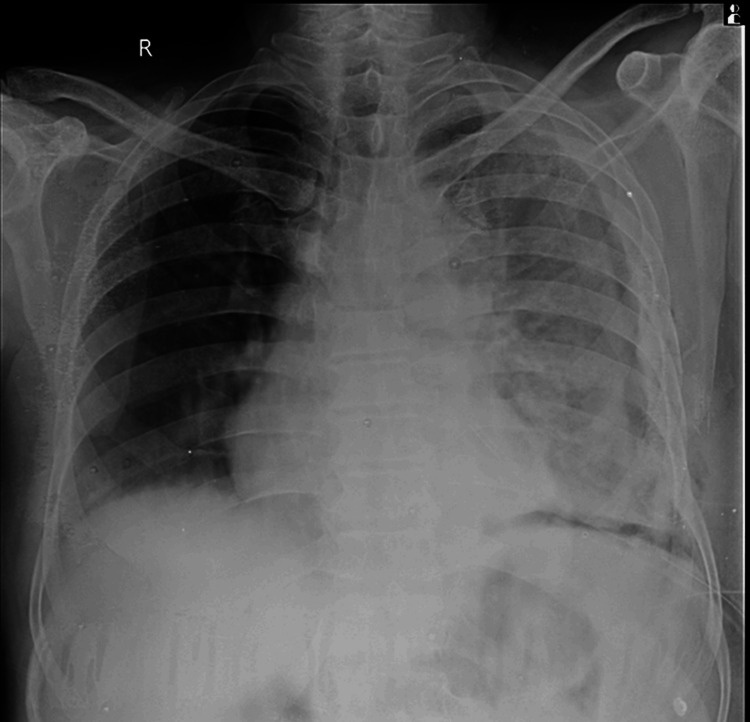
Chest X-ray on discharge post-operative day 10 showing good lung expansion on the left side

The patient demonstrated significant symptomatic improvement. The post-operative drain was removed on day 10, and the patient was discharged from the hospital. Upon discharge, the patient's chest X-ray revealed satisfactory lung expansion on the affected side, as seen in Figure 6.

**Figure 7:**
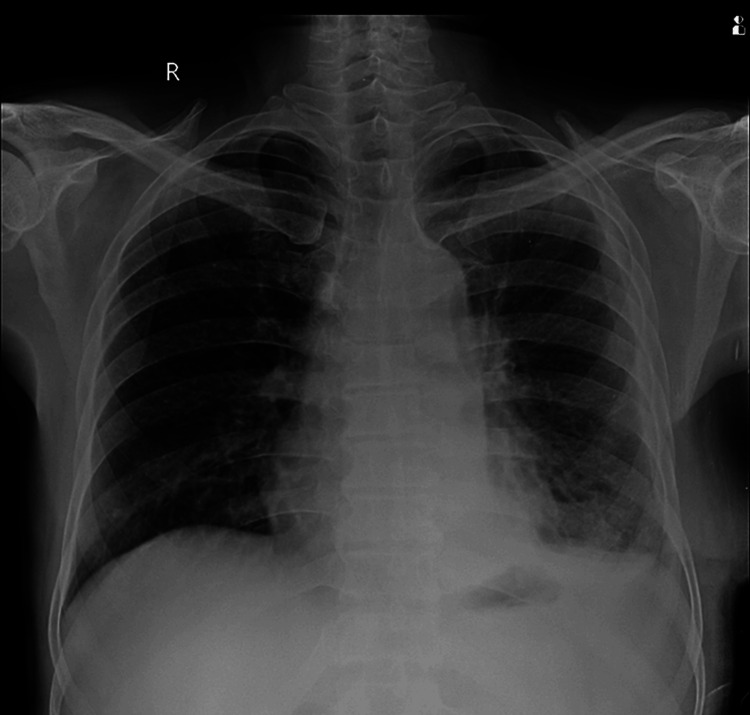
Chest X-ray post-operative day 14

**Figure 8:**
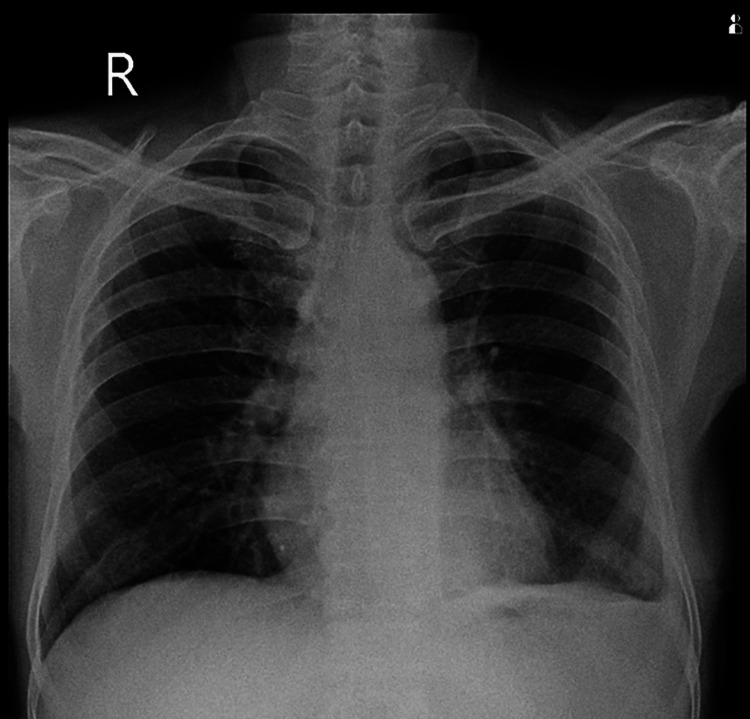
Chest X-ray post-operative day 45

**Figure 9:**
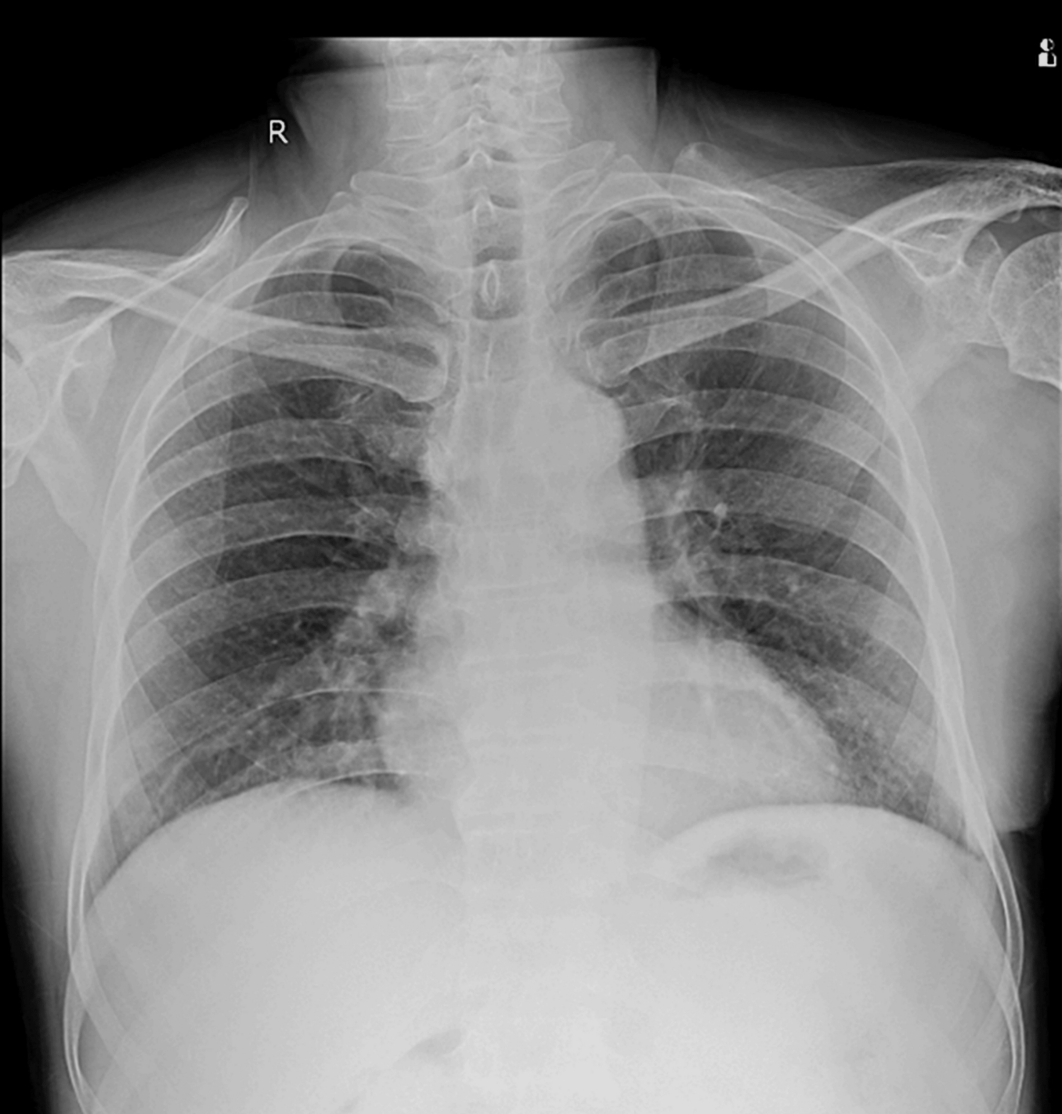
Chest X-ray six months post-operative showing complete resolution

The prescribed medications on discharge included Category-1 Directly Observed Treatment, Short-course (DOTS) anti-TB therapy (AKT) containing isoniazid 300 mg, rifampicin 600 mg, pyrazinamide 1,200 mg, ethambutol 800 mg for six months along with calcium supplementation (500 mg once daily), multivitamins for 14 days, and aceclofenac with serratiopeptidase for seven days. Follow-up chest X-rays on day 14 and day 45 and after six months show gradual improvement and near-complete resolution by the six-month post-operative period (Figures 7-9, respectively).

The patient demonstrated significant symptomatic improvement, as evidenced by radiological improvement, improved exercise tolerance, weight gain, and afebrile status. These improvements not only led to near-complete resolution but also had a profound impact on the patient's quality of life. In a country like India, which has a high TB disease burden with incidence rates of 210 per 100,000 population and TB infection prevalence of 41%, this case report highlights the successful management of a complex pleural disease, resulting in improved outcomes and a reduction in disability-adjusted life years (DALYs).

## Discussion

Our case report highlights significant improvements in patient outcomes following the management of pleural thickening and trapped lung due to TE. The patient experienced better lung expansion, symptom relief, and notable radiological improvements, which demonstrate the effectiveness of our treatment approach. These results are particularly important in regions with a high burden of TB, as they showcase the potential to reduce DALYs and enhance overall quality of life. TE is a rare manifestation of pleural TB characterized by a purulent infection in the pleural cavity with detectable bacilli in the pleural fluid. The American Thoracic Society classifies empyema into three stages: exudative (I), fibrinopurulent (II), and organizing (III) phases [1]. Without proper treatment, TE can lead to serious complications, including pleurocutaneous fistulae, chest wall masses, and rib or bone damage. Risk factors for TE include chronic respiratory diseases, diabetes, cancer, immunosuppression, gastroesophageal reflux disease (GERD), and substance abuse [1]. GERD is a common condition in which the stomach contents move up into the esophagus. Reflux becomes a disease when it causes frequent or severe symptoms or injury.

TE typically presents with pulmonary lesions and purulent pleural fluid, distinguishing it from non-TE with unique clinical features like advanced age and longer illness duration. It often includes lymph node enlargement and pleural nodules, setting it apart further [2]. The course of TE is often complicated by fibrocavitary parenchymal disease and bronchopleural fistulae, making management more challenging [3]. Diagnosis is based on characteristic CT findings, including calcified pleura and purulent fluid, often confirmed by detecting acid-fast bacilli in smear samples. Treatment involves anti-TB medications, drainage procedures, and intrapleural agents to address fibrinolysis [1].

In our case, the patient presented with progressive breathlessness and orthopnea over two months. Physical examination revealed rapid heart and breathing rates and decreased breath sounds on the left side. Diagnostic thoracentesis confirmed exudative effusion positive for CBNAAT *M. tuberculosis*, aligning with findings from studies by Malhotra et al., Kundu et al., and Alioke et al. [2-4,6]. The development of pleural thickening and trapped lung after TE complicated the diagnosis and necessitated surgical intervention. Although the diagnosis was straightforward, managing the condition required extensive lung decortication and a multidisciplinary approach.

The treatment protocol followed standard guidelines, including intravenous antibiotics, anti-TB drugs, and surgical procedures like lung decortication. Similar strategies have been supported in studies by Lee et al. and Long et al., highlighting the effectiveness of antibiotic therapy and pleural drainage. Additional supportive measures, such as calcium supplements and pain relief medications, were also used to improve symptoms [5,7].

The patient’s successful weaning off mechanical ventilation and improved lung expansion on follow-up chest X-rays marked key milestones in their recovery. Previous research by Alioke et al. has linked successful discontinuation of mechanical ventilation with better long-term outcomes, underscoring the significance of these improvements [4]. The absence of complications and the observed lung expansion were positive outcomes in this case.

In India, TB remains a major health issue, with a prevalence of 5.05 per thousand across all forms of TB and an annual incidence of 84 per 100,000 smear-positive cases. A study by Goodchild et al. reported a decline in TB incidence and mortality rates from 1990 to 2019, which helped prevent 1.3 million deaths and 29.2 million DALYs [8,9]. However, TB control programs in low-income countries face challenges like insufficient funding and resources, which hinder disease management and limit access to surgical interventions like decortication. Research by Borgdorff et al. suggests that the DOTS is the most cost-effective intervention, costing between $5 and $40 per DALY gained, compared to less cost-effective alternatives [10].

This case report is limited by its retrospective design and lack of a control group, which may affect the generalizability of the findings. Further longitudinal studies are needed to validate these results, with a focus on preclinical research to develop animal models and explore genetic factors through human genome scans [11].

## Conclusions

This case report demonstrates the effectiveness of a multidisciplinary approach in managing pleural thickening and trapped lung caused by TE. By combining anti-TB medications with surgical intervention, including lung decortication, we achieved significant improvements in the patient’s symptoms, lung function, and overall quality of life. Early diagnosis and comprehensive care, involving collaboration between pulmonologists, thoracic surgeons, and infectious disease specialists, were critical to preventing long-term disability and restoring lung function.

This team-based approach not only addressed the infection but also improved lung mechanics, leading to enhanced daily functioning. These outcomes highlight the importance of timely intervention in regions with a high TB burden. By employing effective management strategies for TB complications, we can reduce the overall disease burden and improve public health, particularly in resource-limited settings.
